# Microstructure and Tribological Performance of Mesocarbon Microbead–Silicon Carbide Composites

**DOI:** 10.3390/ma12193127

**Published:** 2019-09-25

**Authors:** Xiaojie Wang, Xiumin Yao, Hui Zhang, Xuejian Liu, Zhengren Huang

**Affiliations:** 1 Structural Ceramics Engineering Research Center, Shanghai Institute of Ceramics, Chinese Academy of Science, Shanghai 201899, China; zhanghui@mail.sic.ac.cn (H.Z.); xjliu@mail.sic.ac.cn (X.L.); 2 School of Physical Science and Technology, Shanghai Tech University, Shanghai 201210, China; 3 University of Chinese Academy of Sciences, Beijing 100049, China

**Keywords:** microstructure, tribological properties, wear mechanism, MCMB–SiC composites

## Abstract

Mesocarbon microbead–silicon carbide (MCMB–SiC) composites with 0–30 wt % MCMBs were prepared by pressureless sintering (PLS) method at 2200 °C in Ar. The microstructure and tribological properties of the prepared composites were investigated. The results show that there was a finer grain size of SiC with the increase in MCMB content because MCMBs hinder the growth of SiC grains. The hardness of the composites decreased with increasing MCMB content, whereas the fracture toughness fluctuated showing a complex trend. The tribological properties of the composites under dry friction conditions were evaluated using the pin-on-disk method against a SiC counterpart. We found that the tribological properties of the samples were influenced by the oxide film or lubricating film that formed during the wear process on wear surfaces. Different wear mechanisms were found to be associated with differing MCMB contents.

## 1. Introduction

Due to its good resistance against wear, radiation, and corrosion as well as its high-temperature capability and moderate strength, silicon carbide (SiC) is considered to be an excellent structural material and has been applied in many important industries [1,2,3], such as the nuclear and aerospace industries [4,5,6]. In addition to structural applications, SiC ceramic is preferred for tribological applications such as in mechanical seals and bearings [7]. In these friction systems, dry friction or marginally lubricated situations may occur, which require the material to have improved tribological performance. Previous studies have revealed that when SiC ceramic is used as a mechanical sealing material to form self-matched friction pairs in water or oil, the friction coefficient (μ) is very low. The reason for this finding is that SiO_2_ phases are produced during the sliding process on the seal working surfaces of SiC samples, forming a silicic acid film, which is supposedly effective in decreasing friction [8,9]. However, when SiC ceramic is used as the friction part under dry friction or marginally lubricated conditions, their μ is very high, often 0.6–0.7 [7,10]. A high μ leads to the wearing or breaking of parts. Therefore, the tribological performance of SiC ceramic under dry friction needs to be improved to meet increasing demands. Many efforts have been made to reduce the μ of SiC ceramic. Agarwal et al. [11] fabricated carbon fiber–silicon carbide (C_f_–SiC) composites with different C_f_ contents and found that an increase in C_f_ content to 30 wt % can cause μ dorp to 0.1–0.2. Borrell et al. [12] reported that by adding 50 vol % carbon nanofibers (CNFs) into the SiC matrix, the dry friction coefficient can be reduced to around 0.2 despite the low density. Llorente et al. [13] studied the tribological performance of graphene/silicon carbide. The results showed that for the samples with 20 vol % graphene nanoplatelets (GNPs), the wear resistance can improve by 70% compared with monolithic SiC. Other studies have proven that introducing a C phase can effectively improve the tribological performance of SiC materials [12,13,14].

As a new carbon source, mesocarbon microbeads (MCMBs) have recently attracted attention as an active electrode material in lithium and sodium cells due to their excellent electrochemical properties [15,16]. Due to their good self-sintering ability, easy graphitization, and special structure—which is a regular sphere assembled by aromatic layers—MCMBs have been studied as precursors to graphite artifacts [17,18,19]. According to previous reports, MCMBs can graphitize when sintered at high temperature [17,20], possessing similar properties as graphite, such as good lubrication and good thermal shock resistance. 

Some researchers recently demonstrated that MCMBs have many positive effects on silicon carbide. Safi et al. [21] produced graphite–silicon carbide (G–SiC), carbon/carbon–silicon carbide (C/C–SiC) and mesocarbon microbeads–silicon carbide (MCMB–SiC) composites using the liquid silicon infiltration (LSI) method. The results showed that compared with the other composites, MCMB–SiC composites have higher strength and better anti-ablation properties and are promising candidates for aerospace applications.

However, in previous studies, MCMB–SiC composites have mostly been fabricated using the LSI method, in which the MCMB content was unclear, and the tribological properties were not studied. Pressureless sintering (PLS) is one of the commonest fabricating methods for SiC ceramics. Using this method, SiC ceramics can be densified without applied pressure in an inert atmosphere such as Ar [22,23,24]. Using this method, products with a complicated shape and large sizes can be produced. The compositions of samples can also be designed as needed.

In this work, we prepared MCMB–SiC composites with different MCMB contents via PLS method and investigated the tribological behaviors and microstructure of the composites with different MCMB contents. The wear mechanisms of the samples were also discussed in detail.

## 2. Experimental Procedures

### 2.1. Material Fabrication

Commercially available MCMBs with an 8–10 μm particle size and submicrometer SiC powders (D_50_ = 0.5 μm) were used in this work. Boron carbide (B_4_C) powders (D_50_ = 1.5 μm) were used as sintering aids. Firstly, phenolic resin was dissolved into ethanol to obtain a mixture solution, and then the sintering aids and SiC powders were added into the solution. After two h of ball milling with SiC balls, MCMBs were added into the suspensions and milled for another two h, followed by solvent evaporation. The mixed powders were cold-isostatic-pressed at 100 MPa to form green bodies. After pyrolysis of the phenolic resin under an argon atmosphere, the green bodies were sintered at 2200 °C in Ar for 1 h. The prepared samples with 0, 5, 10, 15, 20, and 30 wt % MCMBs were labeled M-0, M-5, M-10, M-15, M-20, and M-30, respectively.

### 2.2. Materials Characterization

The bulk densities of the samples were measured using Archimedes’ principle. The theoretical densities of each sample were calculated according to the rule of mixtures. The theoretical densities of SiC and MCMBs applied in our calculation were 3.20 and 2.16 g/cm^3^, respectively.

Microstructure evolution was investigated using field emission scanning electron microscopy (FE-SEM; S-4800, Hitachi, Tokyo, Japan). Transmission electron microscopy (TEM; JEM-2100F, JEOL, Tokyo, Japan) was used for the detailed study of grain boundaries at higher magnification.

Fracture toughness (K_IC_) tests were conducted on a universal testing machine (Instron-5566, Instron Co., Boston, MA, USA) using the single-edge notched beam method with a span of 24 mm and groove depth of 3 mm. The samples used for toughness tests were machined into dimensions of 3 mm × 6 mm × 30 mm. Five samples were tested for each of the different values of MCMB content. The hardness tests were conducted on a Vickers hardness tester (TUKON-2100B, Instron Co., Boston, MA, USA) with a load of 0.5 kg.

Tribological properties were tested using a standard pin-on-disk wear tester (DX-NPO1, CSM Instruments SA, Peseux, Switzerland) with a load of 20 N at a speed of 200 rpm. Each test was conducted for 60 min. The samples were machined to disks with a size of Φ 25 mm × 6 mm and polished. The counterparts used in the tests were SiC pins with a Φ 3 mm hemispherical end. The wear rates were measured using a white light interferometer (Contour GT-K, Bruker Optics, Inc., Billerica, MA, USA). The worn surfaces of the samples were examined using SEM (ProX-SE, Phenom-World, Eindhoven, Netherlands).

## 3. Results and Discussion

### 3.1. Initial Powders

**Figure 1:**
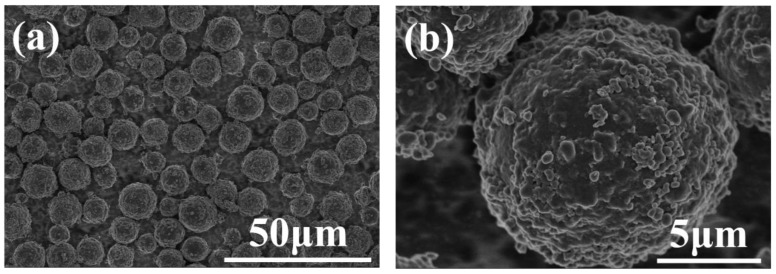
Morphology of mesocarbon microbeads (MCMBs) at (**a**) low and (**b**) high magnification.

The MCMB morphology is shown in Figure 1. The MCMBs were regular spheres. From the high magnification picture (Figure 1b), every sphere was assembled with many flaky components which, according to previous reports, are flaky aromatic molecules [20,25].

### 3.2. Microstructure and Density

**Figure 2:**
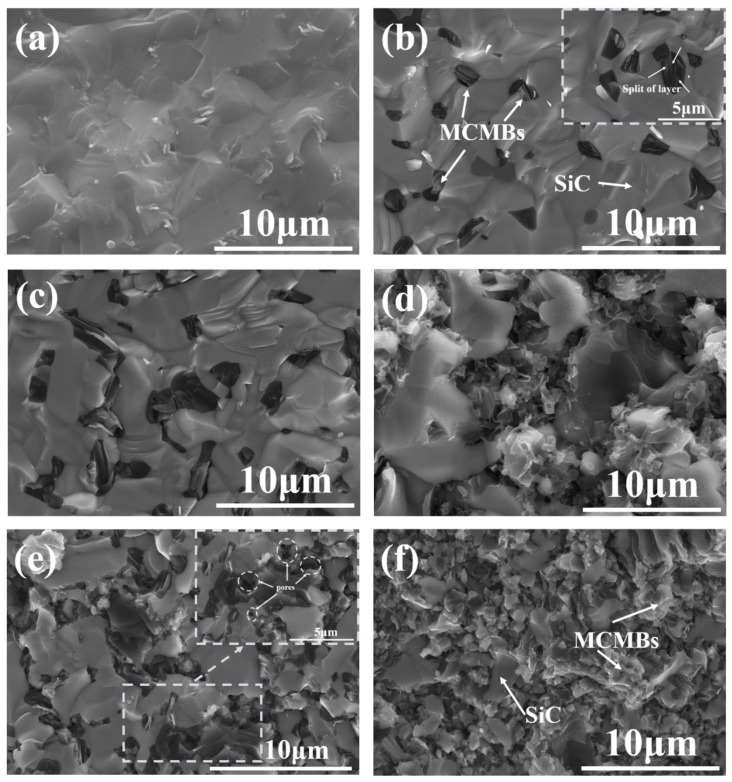
Microstructure (fracture surfaces) evolution of MCMB–SiC composites as MCMB content increases from 0 (M-0) to 30 wt % (M-30): (**a**) M-0; (**b**) M-5; (**c**) M-10; (**d**) M-15; (**e**) M-20; (**f**) M-30.

**Figure 3:**
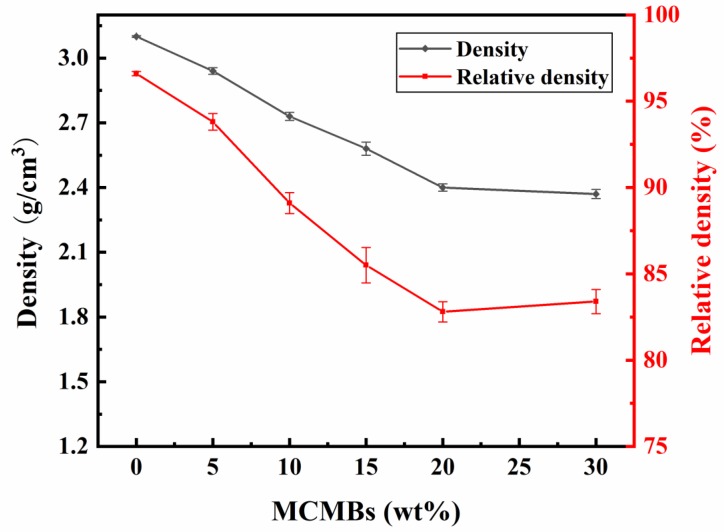
Density and relative density fluctuate with increasing MCMB content.

Figure 2 shows SEM images of the fracture surface microstructure of the composites. In the samples, black particles are sintered MCMBs, and the gray matrix is SiC. As can be observed, for monolithic SiC (M-0, Figure 2a), the microstructure was rather dense. The For MCMB–SiC composites (Figure 2b–f), the MCMBs were homogeneously distributed in the SiC matrix. As the MCMB content increased, the size of the SiC grains decreased due to the impeding mechanism of MCMBs, which is in agreement with the function of carbon in the solid-state sintering of SiC ceramics [26]. The agglomeration of MCMBs simultaneously increased. After being sintered at 2200 °C in Ar, the shape of MCMBs collapsed from initial regular spheres to irregular particles, and the particle size of the MCMBs shrank from the initial size of 8–10 μm to the final scale of 1–2 μm. During the sintering process, gaseous products such as H_2_, CH_4_, CO_2_, and H_2_O were released, accompanying the carbonization process and with significant volume shrinkage [27]. In M-5 (Figure 2b) samples, MCMBs particles were dispersed in the SiC grain boundaries and surrounded by coarse SiC grains. When the MCMB content increased to 10 wt %, two or more MCMBs particles stuck together and grew into larger particles. Some of those particles were squeezed into elongation. In the M-5 and M-10 (Figure 2c) samples, MCMB particles were isolated from each other by SiC grains, whereas in M-15 (Figure 2d), MCMB particles started to connect and formed flimsy conjunctions. Simultaneously, the SiC matrix was prone to separation by MCMBs, and the SiC grains became finer as the MCMB content increased. In M-20 (Figure 2e) and M-30 (Figure 2f), an appreciable amount of MCMBs aggregated, and pores were observed. The contact area between SiC grains was significantly reduced. MCMBs have lower density than SiC, so the density of both sharply decreased as the MCMB content increased. The relationships between MCMB content and densities and relative densities of the composites are depicted in Figure 3. With increasing MCMB content, the density of the composites sharply reduced from 3.10 to 2.37 g/cm^3^, due to the low density of MCMBs and lower densification. In this article, the monolithic SiC was densified through PLS method using B and C as the sintering aids; during sintering, B can form a solid solution with SiC to reduce the grain boundary energy [28], and C can remove the oxide film around SiC grains to increase the surface energy of SiC thereby promoting sintering [29]. The C in MCMBs also have the same effect on motiving the sintering of SiC. However, when there are excessive C, the densification of SiC can be hindered. The relative densities of the samples firstly decreased as the MCMB content increased, then increased slightly (Figure 3). The minimum value of 82.8% was observed for M-20. Such a sharp decrease in the relative densities may be caused by the volatilization of hydrogen [30] and the hindering effect of MCMBs. Release of gas significantly increased porosity in the composites, and the shrinkage of the MCMBs was not enough to fill the pores. As shown in Figure 2f, after sintering, the aromatic layers of MCMBs were split; the split also hindered the densification process. However, the relative density of M-30 was a little higher than that of M-20, which was due to the self-sintering property of MCMBs [19,31]. As shown in Figure 2, the phase distribution of M-30 was MCMB-dominant during sintering; the shrinkage of MCMBs can fill some pores. However, the relative density of M-30 was still low. Therefore, densification of the composites with high MCMB content would still be challenging. 

### 3.3. Mechanical Properties

**Figure 4:**
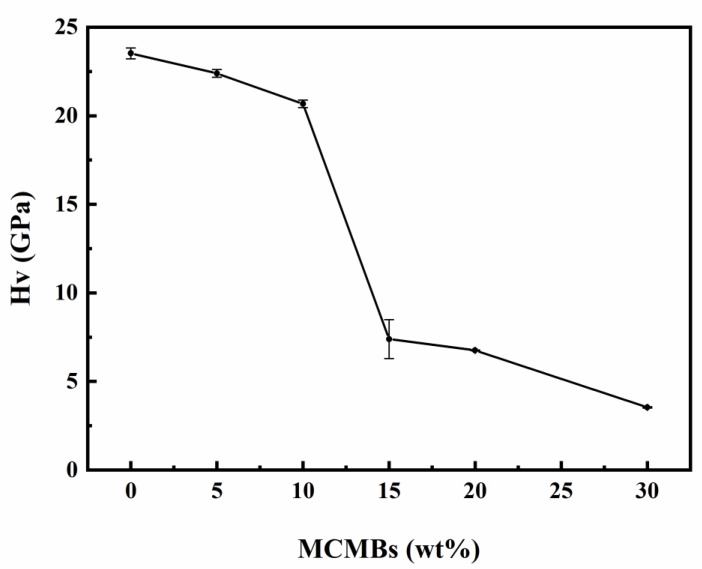
Relationship between MCMB content and hardness (HV).

With the increasing of soft MCMB phase, the hardness (Figure 4) of the composites decreased sharply from 23.5 to 3.5 GPa. When the MCMB content was still below 15 wt %, the SiC phase played a dominant role in the composites. Therefore, the composites had higher hardness. After this point, the effect of increasing MCMBs led to their gradually dominating the properties of the composites, which led to lower hardness.

**Figure 5:**
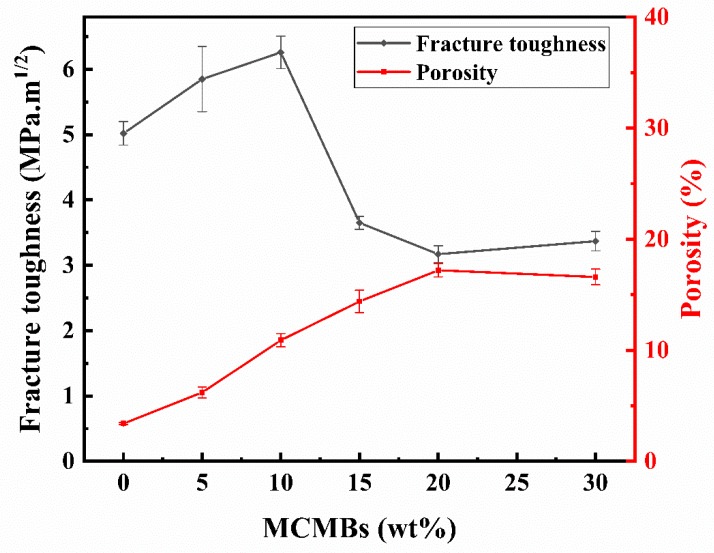
Fracture toughness and porosity fluctuate with MCMB content.

As shown in Figure 5, the fracture toughness of the samples fluctuated with the increase in MCMB content. First, the curve increased, and the maximum value was obtained with M-10 samples. This was followed by a sharp drop, with the minimum value observed with M-20 samples. However, when the addition of MCMBs reached to 30 wt %, the toughness of the samples began to slightly increase. 

**Figure 6:**
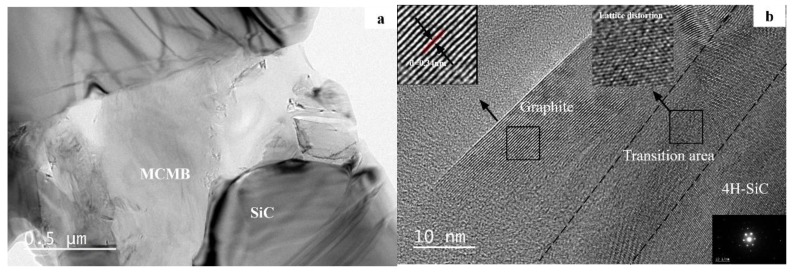
(**a**) TEM and (**b**) high resolution TEM (HRTEM) images of sample M-10.

**Figure 7:**
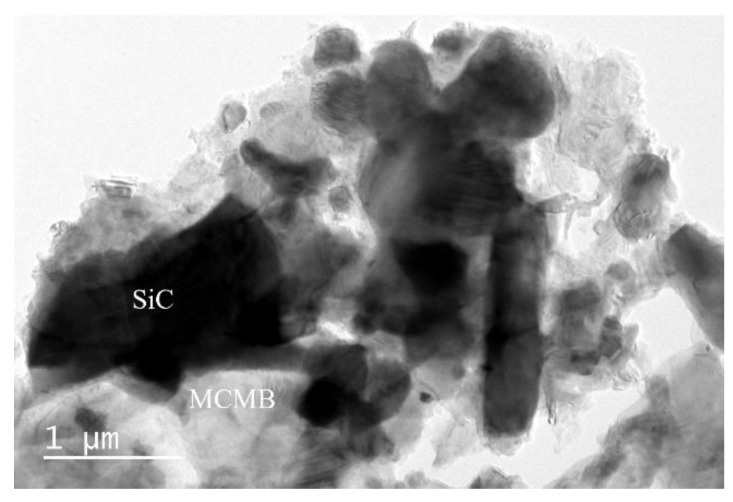
TEM image of sample M-20.

When the MCMB content in the samples increased to 10 wt %, the toughness of the samples reached a maximum, at about 6.26 MPa·m^1/2^, which can be explained by the dispersion toughening mechanism. A certain number of second-phase particles can be introduced to absorb the energy of cracks by obstructing the growth of cracks or making cracks deflect, then leading to a higher toughness. In our research, when the second-phase content reached 10 wt %, a superior toughening effect was observed. With the increase in MCMB content, pores in the samples increased and the structure loosened (Figure 2), thus leading to a sharp drop in toughness, which was 3.17 MPa·m^1/2^ for sample M-20. The porosities of the samples are also displayed in Figure 5. It can be seen that when the MCMB contents were ≤10 wt %, the fracture toughness of the samples increased with the increasing porosity of the samples. With the increasing MCMB contents, the particle sizes of SiC grains decreased (seen in Figure 2), which declined the deterioration effect of increasing pores on the fracture toughness. However, when the MCMB content was more than 10 wt %, when the porosity increased, the fracture toughness dropped; when the porosity decreased, the fracture toughness increased again. To further reveal the microscopic mechanism, TEM analyses were conducted. TEM observations of M-10 (Figure 6a) showed that grain boundaries between SiC grains and MCMBs were clean, with no third phases. The high resolution TEM (HRTEM) image of M-10 (Figure 6b) revealed more details: The MCMB particles were partially graphitized, and the interlayer spacing was around 0.34 nm, which is a typical for graphite structure. The detected SiC grains were in the form of 4H–SiC. At the interfaces between MCMBs and SiC grains, the graphite layers and the SiC crystal lattices partially overlapped. At the junction of the two phases, lattice distortion of SiC was observed. Lattice distortion indicated that the graphite phase interacted with the SiC phase at the lattice level, which provides evidence that these two phases formed strong interface bonding. The lattice distortion can also increase the fracture energy. Thus, the fracture toughness of M-10 was the highest of the samples in our research. However, for M-20, MCMBs distributed in the SiC matrix started to form a net structure and separated the SiC grains into small island structures (Figure 7), and its rather high porosity (Figure 5) also hindered the growth of SiC grains. The separation effect weakened the bonding strength among SiC grains, and the MCMBs are not able to form strong junctions because the contact area was limited. These two kinds of weakening effects led to deterioration of the mechanical properties of M-20. As the proportion of MCMBs increased further, the materials gradually transitioned from SiC-dominant to MCMB-dominant. When the MCMB content increased to 30 wt %, the SiC–SiC grain boundaries decreased and the MCMB–MCMB boundaries increased. Due to self-sintering of MCMBs, the MCMB–MCMB bonding strength was enhanced; therefore, the fracture toughness slightly increased.

### 3.4. Tribological Performance under Dry Friction Condition 

#### 3.4.1. Friction Coefficient 

**Figure 8:**
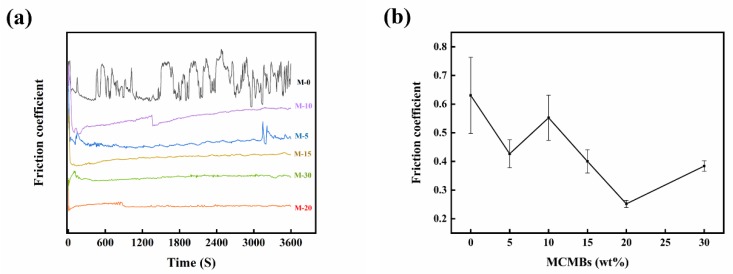
(**a**) Friction coefficient evolution as a function of the sliding time; (**b**) relationship between MCMB content and friction coefficient.

The friction coefficients of the samples with different MCMB contents are shown in Figure 8. For monolithic SiC (M-0), the dry friction coefficient (µ) fluctuated widely between 0.5 and 0.75, which is close to the reported value of 0.6–0.7 for solid-state sintering-SiC materials (carbon content 3–6 wt %) under dry sliding conditions [10]. The fluctuation of µ might be caused by the formation and removal of oxide film. A running-in time could be observed in the friction curves of all samples, M-5 to M-30. After the running-in period, the curves fluctuated with differing trends. For sample M-5, µ was about 0.43. The friction curve fluctuated slightly with increasing sliding time; however, at the end of the sliding test, the curve started to fluctuate visibly, probably due to the peeling of oxide film. Exfoliated oxides may cause abrasive wear; therefore, the friction coefficient became unstable. For sample M-10, µ increased to 0.55 and its friction curve was typical. After the running-in stage, µ first gradually increased with the sliding time and was relatively steady for a period of time; suddenly, the curve dropped obviously, and after that, µ increased again with the same trend as in the period before the transition point. The variation trend in µ indicated a transition in wear mechanism potentially accompanied by severe wear behavior [32,33]. For sample M-15, the friction coefficient value decreased to around 0.4, and the friction curve remained steady throughout the testing stage. When MCMB content increased to 20 wt %, µ dropped to around 0.25. As with sample M-10, the friction curve of M-20 also increased toward a transition point, but the difference was obvious. For sample M-20, before the transition point, the friction curve maintained an upward trend, but after that, the curve showed a downward trend. Different trends may indicate differences in wear behaviors and wear mechanisms. The improved tribological behaviors of samples M-15 and M-20 could be caused by the lubricating effect of sintered MCMB particles. During the dry sliding process, soft sintered MCMB particles were firstly worn, followed by the particles being ground into slices due to their special layer structure. Under the pressure of the load, those slices tiled across the whole working surface to form a lubricating film, as has been reported by other researchers [34]. As the film thickened, the coefficient decreased, and the wear became mild. With the increase in MCMB content, the bonding strength between particles weakened; the wear of sintered MCMBs intensified, and the lubricant film considerably thickened. However, as the content of MCMBs reached 30 wt %, the µ of the samples increased again due to incompact microstructure and slightly improved mechanical properties. During the sliding tests, some hard SiC particles were worn and fell onto the friction surface, then broke the lubricant film formed by MCMBs. Therefore, the friction coefficient of sample M-30 was even higher than that of sample M-20.

#### 3.4.2. Wear Rate

**Figure 9:**
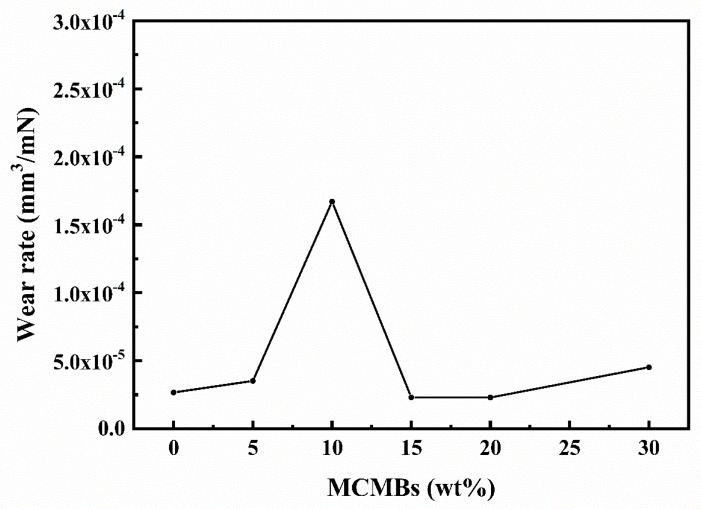
Relationship between MCMB content of the samples and wear rate.

As shown in Figure 9, the wear rate of sample M-10 (1.67 × 10^−4^ mm^3^/Nm) was almost one order of magnitude higher than that of the other samples. Such severe wear may be related to the wear transition mentioned above in the friction coefficient (Section 3.4.1). For other samples, although the MCMB content was different, the differences in their wear rates were not significant. However, the reasons for their good wear resistance differ. When the MCMB content was under 10 wt %, the hardness of the samples was rather high, and the bonding strength of the grains was strong, and thus, the wear rate was low. When the MCMB content increased to more than 10 wt %, the hardness dropped dramatically. The wear rates of the samples are associated with the wear of MCMBs, which can form a lubricating film on the working surface and then prevent the matrix material from being further worn. As for sample M-30, the SiC grains were separated by MCMBs particles, and the interfaces between SiC grains were weak; thus, during the sliding process, SiC grains were worn and the hard SiC particles might have caused abrasive wear.

#### 3.4.3. Wear Mechanism

**Figure 10:**
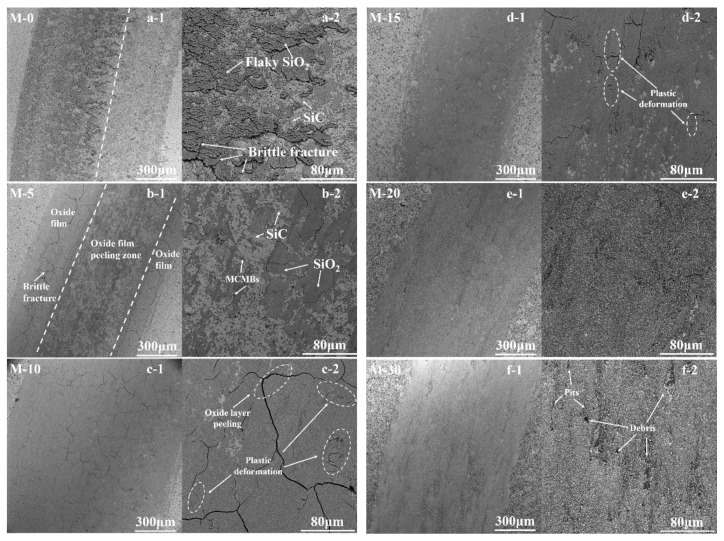
Worn surface morphologies of the samples; (**a-1**, **a-2**) M-0, (**b-1, b-2**) M-5, (**c-1, c-2**) M-10, (**d-1**, **d-2**) M-15, (**e-1**, **e-2**) M-20, and (**f-1**, **f-2**) M-30.

The worn surface morphologies of samples after sliding tests were investigated using SEM, as shown in Figure 10. By combining the several different kinds of wear patterns with the friction coefficient and wear rate, we found different wear mechanisms among the samples with different MCMB contents.

**Figure 11:**
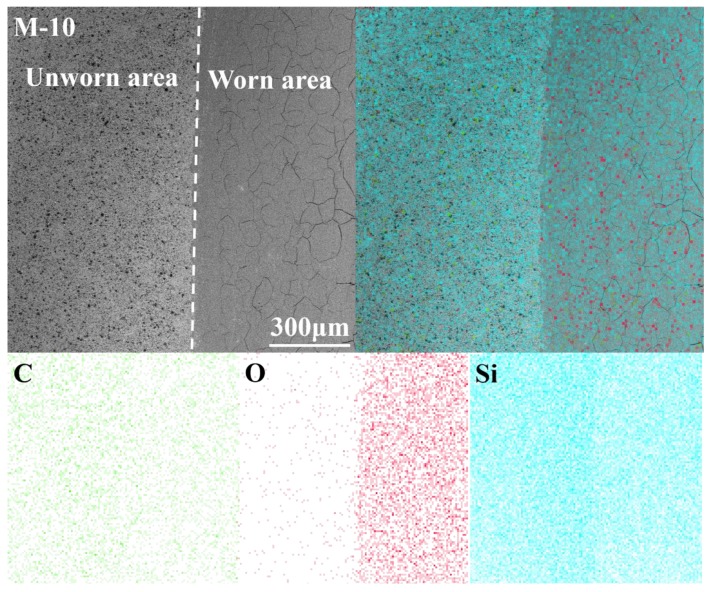
Energy dispersive spectrometer (EDS) images of sample M-10.

When discussing wear mechanisms of SiC, mechanical wear (microcracks), abrasive wear, and tribochemical reactions (oxide layers) are always included [35,36]. In this research, these mechanisms were also involved, although the changes in composition resulted in the mechanisms being complex. As can be observed in the SEM images (Figure 10), some microcracks appeared in all wear tracks. These cracks may have been caused by the stress generated by the load and friction force on the contact surfaces as well as the thermal expansion mismatch between the surface materials and matrix materials. Elemental analysis (Figure 11) showed that oxygen concentrations in the worn area were higher than in the unworn area, which suggested that a tribochemical reaction occurred during the sliding process and that an oxide film formed on the worn area.

For samples M-0 and M-5, (Figure 10a,b), the wear tracks were uneven, which was caused by the inhomogeneous distribution of SiO_2_ film (oxide film). Brittle fractures (micro-cracks) can be observed on the wear tracks; most of these fractures occurred on the oxide film. For samples M-0 and M-5, the hardness was close to the friction pair (SiC pin), which indicates that the bonding strength between the SiC grains was rather strong. During the sliding process, the pull-out of SiC grains was very hard; thus, the wear was mostly caused by removal of the oxide film. Due to their high hardness, the oxide film was tightly bonded with the matrix. Under friction force and load, brittle fracture was directly generated without any deformation process. Therefore, for samples M-0 and M-5, the wear mechanisms were dominated by the brittle fracture of the oxide film. 

For sample M-10, the worn surface was completely covered by an oxide film full of micro-cracks. Form the high magnification picture (Figure 10c-2), some plastic deformations can be observed. Simultaneously, in some areas, the cracked oxide film had a tendency to peel off. The wear track of M-10 looked smooth, and the friction coefficient and wear rate were rather high. Combining its high friction coefficient with a transition point, exceptionally high wear rate, and high fracture toughness, the following wear mechanism of sample M-10 was determined. Before the transition point, there was a constant and slight increase in the friction coefficient, which indicated the formation of an oxide layer. During this period, the SiC grains and SiO_2_ film at the sliding surface were pulled by the friction force, which led to the generation of tensile stress on the sliding surface. Due to its high-fracture toughness, the stress continued to accumulate. Under the friction force and load, plastic deformations generated on the oxide film, which might have resulted in the final peeling of the film and also caused the sudden drop in the friction coefficient. After the transition point, the friction coefficient increased again, which might suggest it was undergoing the same process. As the duration of plastic deformation reached the transition point, the wear mechanism of sample M-10 was therefore closely related to the generation and peeling of the oxide film. 

For M-15, the wear tracks were smooth and shallow. The oxide film did not completely cover the worn surface. Some micro-cracks and plastic deformations were also observed, but the amount of cracking was much less than with sample M-10. The friction coefficient curve was smooth, and the wear was mild. During the sliding test, due to its low hardness, the MCMB particles were first detached from the matrix and were then ground to flat species that could form a lubricating film on the sliding surface; thus, the tribological performance of sample M-15 was improved compared to that of sample M-10. Therefore, in our present work, the wear mechanism changed from oxide-film-dominated to lubricating-film-dominated with the increase in MCMB content. 

In this research, the wear resistance of sample M-20 was even better than that of pure SiC. The wear track was rather smooth, and only a few micro-cracks were observed. The sliding process was stable, and as the sliding time increased, the friction coefficient presented a descending trend. This good tribological performance may be due to the lubricating film that was formed by sintered MCMB particles. From the high magnification picture, the micro-cracks were thin and filled by some microparticles which may have been formed by MCMBs. During the sliding process, the sintered MCMBs were pulled out and then ground into small sheets, and some of the sheets spread out on the surface to form a lubricating film. Some of them also filled the micro-cracks to smoothen the wear track, such that the wear surface was given the ability to self-heal from wear damage. Thus, for sample M-20, the wear mechanism mainly involved the generation of a lubricating film formed by sintered MCMBs.

For sample M-30, the wear track was generally smooth and almost devoid of micro-cracks. Nevertheless, some scratches and pits were observed on the wear track. These defects may have been caused by the SiC particles that were pulled out during the sliding process as a result of the weak bonding between the particles. Hard SiC particles scratched the lubricating film, causing abrasive wear. Thus, the friction coefficient of sample M-30 fluctuated slightly, and the wear rate of sample M-30 was higher than that of M-20.

To summarize, in this study, the wear mechanism of all samples can be divided into two categories: Oxide-film-dominated or lubricating-film-dominated. For samples with low MCMB content (≤10 wt %), the tribological performance was closely related to the formation and removal of oxide film; when the MCMB content increased to more than 10 wt %, the tribological behavior was more associated with the thickness and roughness of the lubricating film.

## 4. Conclusion

In this study, MCMB–SiC composites containing 0 to 30 wt % MCMBs were fabricated using a pressureless sintering process. We found that finer SiC grains are observed with the increase in MCMB content. The densities of the samples decrease with increasing MCMB contents, while, the fracture toughness of the samples fluctuate widely. Sample 10-M is the toughest, which may be caused by the toughing effect of MCMBs phases. The tribological properties were measured, and the wear mechanisms were studied. The results show that the tribological performance of MCMB–SiC composite can be effectively improved by adding 20 wt % MCMBs. Such a good self-lubricating performance may be caused by the lubricating effect of MCMBs. We also found that at low MCMB contents, the wear mechanisms are dominated by the formation and removal of oxide films on the working surface, whereas at high MCMB contents, the wear mechanisms of the composites are dominated by the thickness and roughness of the lubricating films. The evolution of mechanical properties, tribological properties, and wear mechanisms indicates that by adjusting the composition, the MCMB–SiC composites may have the potential to be suitable for different applications.
